# Water awareness and bioethical perspectives among medical students at Mersin University Faculty of Medicine: an analytical cross-sectional study comparing academic-stage groups

**DOI:** 10.3389/fmed.2026.1917669

**Published:** 2026-09-03

**Authors:** Selda Okuyaz, Oya Ögenler

**Affiliations:** Department of History of Medicine and Ethics, Faculty of Medicine, Mersin University, Mersin, Türkiye

**Keywords:** bioethics, ecological physicians, medical education, medical students, water consumption

## Abstract

**Background:**

The escalating environmental crisis underscores water as a vital bioethical responsibility and public health determinant. Accordingly, water consumption, sustainability, and related ethical responsibilities are relevant topics in medical education.

**Objective:**

This study aimed to compare water-consumption behaviours, awareness, knowledge, and attitudes toward water use and water-related ethics between Year 3 and Year 6 medical students.

**Methods:**

This analytical cross-sectional study was conducted at Mersin University Faculty of Medicine, Türkiye, and compared two independent cohorts of medical students (Year 3 and Year 6) to evaluate their knowledge and attitudes toward water consumption from a bioethical perspective. The findings therefore represent cross-sectional associations between academic stage and the measured outcomes in two independent cohorts. Data were collected using a self-administered questionnaire incorporating the Water Consumption Behaviour Scale. Descriptive statistics, chi-square tests, and independent-samples t-tests were used for the analyses.

**Results:**

A total of 246 medical students participated in the study. Correct-response rates were higher among Year 6 students than Year 3 students for recommended daily water intake (26.3% vs. 7.4%, *p* < 0.001) and water-conservation practices (61.4% vs. 44.4%, *p* = 0.025). No significant gender differences were observed in knowledge levels. Water Consumption Behaviour Scale scores differed by academic stage: Year 6 students had higher Water Awareness and Personal and Social Responsibility scores, whereas Year 3 students had higher Water Consumption, Water Pollution, Water Management, and total scores (*p* ≤ 0.004).

**Conclusion:**

Academic stage was associated with distinct patterns of water-related knowledge and scale scores. Year 6 students had higher correct-response rates for two knowledge items, whereas Year 3 students had a higher total Water Consumption Behaviour Scale score. These findings represent cross-sectional associations between academic stage and the measured outcomes in two independent cohorts. The observed patterns identify water ethics, planetary health, and sustainability as relevant topics for medical curricula.

## Introduction

The Anthropocene's environmental crisis has exacerbated freshwater losses, making water scarcity a primary threat to wellbeing. Driven by climate change and pollution, this crisis has evolved into a 21st-century bioethical challenge (1, 2). Consequently, medical goals are shifting beyond individual care toward a broader perspective that recognises the fundamental interdependence between human health and ecological systems (2, 3).

Beyond infection control, water management within health disciplines is a vital bioethical concern (1). ‘Water ethics' frames fair distribution and conservation as healthcare responsibilities. While traditional bioethics focuses on physician–patient dynamics, contemporary ecocentric approaches emphasize broader obligations (3). Thus, future physicians must view water not just as a clinical input, but as a key community health determinant.

Medical education is a formative period for shaping professional identities and ethical practices. Sustainability is increasingly conceptualized as a moral obligation linked to justice and non-maleficence (4). Consequently, students must understand how ecological and social determinants shape patient and community health (5). Assessing students' knowledge and attitudes toward water conservation provides insight into their future resource stewardship. Furthermore, comparing independent academic-stage groups enables the examination of associations between academic stage and water ethics (6). Within this context, medical schools have a critical responsibility to prepare future physicians who recognize water stewardship as both a public health obligation and a bioethical responsibility. The term “ecological physician” can therefore be used to describe a physician who recognizes the interdependence between human and ecosystem health and considers environmental conditions within the scope of professional responsibility (7). Comparing students at different academic stages may provide insight into the association between academic stage and water-related awareness.

Given the growing importance of planetary health and environmental bioethics in medical education, it is important to examine whether water-related knowledge and ethical awareness differ between students at different stages of medical education. This analytical cross-sectional study was conducted at Mersin University Faculty of Medicine, Türkiye, to compare pre-clinical (Year 3) and internship (Year 6) medical students with respect to water-related knowledge, awareness, attitudes, and water consumption behaviours from a bioethical perspective. Specifically, the study assessed knowledge regarding water consumption and conservation, attitudes measured by the Water Consumption Behaviour Scale, and differences between two independent academic-stage groups.

The aim of this analytical cross-sectional study, conducted at Mersin University Faculty of Medicine during 2022 and 2025, was to compare pre-clinical (Year 3) and internship (Year 6) medical students regarding water-related knowledge, awareness, attitudes, and water consumption behaviours from a bioethical perspective.

The study was designed to address the following research questions:

Do pre-clinical (Year 3) and internship (Year 6) medical students differ in their knowledge of water consumption and water conservation?

Do water awareness, attitudes, and bioethical perspectives differ between the two stages of medical education?

Is academic stage associated with water-related knowledge and environmental bioethical awareness?

## Materials and methods

This analytical cross-sectional study was conducted at Mersin University Faculty of Medicine to compare medical students' knowledge, awareness, and attitudes toward water consumption from a bioethical perspective at two different stages of medical education. Rather than following the same individuals over time, the study compared two independent groups of students: third-year (pre-clinical) students and sixth-year (internship) students.

The pre-clinical group consisted of students enrolled in the third year during the 2021–2022 academic year, whereas the internship group consisted of sixth-year students recruited in 2025. The inclusion criteria were enrolment in the designated academic year at the time of data collection, voluntary participation, and provision of written informed consent. Students who were not enrolled in the designated academic year, did not provide informed consent, or submitted substantially incomplete questionnaires were excluded from the study.

The target population of the pre-clinical group comprised 347 eligible third-year medical students. A *post hoc* sample size assessment indicated that, for a finite population of 347 students, a minimum of 183 participants would be required at a 95% confidence level and a 5% margin of error. The number of participating pre-clinical students exceeded this reference threshold. Nevertheless, the voluntary participation and convenience sampling approach should be considered when interpreting the representativeness of the findings. The target population of the internship group comprised 125 eligible sixth-year medical students. Data collection was conducted during the internship period, when students were distributed across different clinical rotations and hospitals. Owing to clinical duties, rotation schedules, and the voluntary nature of participation, not all eligible students were available during the data collection period. Consequently, 57 internship students agreed to participate and completed the questionnaire. A total of 189 of the 347 eligible pre-clinical students and 57 of the 125 eligible internship students completed the questionnaire, corresponding to participation rates of 54.5% and 45.6%, respectively. Participants were recruited using a convenience sampling approach based on voluntary participation among eligible students who were available during the data collection period.

Data for the pre-clinical group were collected in 2022, whereas data for the internship group were collected in 2025. Thus, the study compared two independent cohorts assessed at different calendar times rather than following the same students longitudinally. During this period, the formal medical curriculum remained unchanged with respect to education on water consumption behaviour, water ethics, and sustainability.

Data were collected using a self-administered questionnaire consisting of two sections. The first section included questions on sociodemographic characteristics (age, gender, academic year) and knowledge regarding water consumption based on World Health Organization recommendations, including recommended daily water intake, drinking water accessibility, and water conservation practices. The second section consisted of the Water Consumption Behaviour Scale, a 16-item instrument designed to evaluate attitudes toward water consumption. The scale comprises five subdimensions: Water Consumption (Items 1, 3, 4, and 6), Water Awareness (Items 5, 8, and 16), Water Pollution (Items 2, 10, and 12), Water Management (Items 7, 11, and 13), and Personal and Social Responsibility (Items 9, 14, and 15). Each item is rated on a five-point scale (1–5), yielding a total score ranging from 16 to 80. The Water Consumption Behaviour Scale consisted of 16 items scored on a five-point Likert scale (1 = strongly disagree to 5 = strongly agree), yielding total scores ranging from 16 to 80. The possible score range for the Water Consumption subscale is 4–20, whereas the Water Awareness, Water Pollution, Water Management, and Personal and Social Responsibility subscales each range from 3 to 15. Higher scores on the Water Awareness subscale indicate greater awareness of water-related issues; higher scores on the Water Consumption subscale indicate more responsible water-consumption behaviours; higher scores on the Water Pollution subscale indicate greater awareness of water pollution; higher scores on the Water Management subscale indicate more positive attitudes toward sustainable water management; and higher scores on the Personal and Social Responsibility subscale indicate stronger perceptions of individual and social responsibility for water conservation. Therefore, higher scores on all subscales and on the total scale consistently represent greater water awareness and more responsible water-consumption behaviours. The original validation study reported a Cronbach's alpha coefficient of 0.83 (8). In the present study, Cronbach's alpha was 0.75, indicating acceptable internal consistency.

Ethical approval was obtained from the Mersin University Clinical Research Ethics Committee (Approval No. 2022/239, April 6, 2022). The study was conducted in accordance with the Declaration of Helsinki. Written informed consent was obtained directly from each participating medical student before data collection.

Statistical analyses were performed using descriptive statistics, chi-square tests, and independent-samples t-tests. Continuous variables were expressed as mean ± standard deviation (SD), and categorical variables as frequencies and percentages. Homogeneity of variances was assessed using Levene's test. Student’s independent-samples t-test was used when equal variances were assumed, whereas Welch’s *t*-test was applied when this assumption was violated. Effect sizes were reported as Cohen’s d, and 95% confidence intervals (95% CI) were calculated for mean differences. Mean differences and effect-size directions were calculated by subtracting the pre-clinical group mean from the internship group mean. Third-year and sixth-year students were analyzed as independent groups. Statistical significance was set at *p* < 0.05.

Because the study was conducted at a single medical school and relied on self-reported data, the findings may be subject to selection bias and social desirability bias, limiting their generalizability. Furthermore, given the analytical cross-sectional design and the comparison of independent student groups, the observed differences represent associations between academic stage and water-related knowledge, awareness, and attitudes.

## Results

The study included 246 medical students: 135 (54.9%) were male and 111 (45.1%) were female; 57 (23.2%) were Year 6 students and 189 (76.8%) were Year 3 students. The mean age of the participants was 21.19 ± 2.32 years.

No statistically significant gender differences were observed in correct responses to the water-consumption knowledge questions. The correct-response rates for daily water-consumption frequency were 59.5% among female students and 49.6% among male students (*χ*² = 2.37, *p* = 0.124). The corresponding rates for recommended daily water intake were 8.1% and 14.8%, respectively (*χ*² = 2.64, *p* = 0.105). Correct-response rates were also similar for knowledge of the recommended walking-distance standard (36.0% vs. 37.0%; *χ*² = 0.03, *p* = 0.871) and water conservation methods (48.6% vs. 48.1%; *χ*² = 0.006, *p* = 0.938). By academic stage, correct-response rates were higher among Year 6 students than Year 3 students for recommended daily water intake (26.3% vs. 7.4%; *χ*² = 15.06, *p* < 0.001) and water-conservation methods (61.4% vs. 44.4%; *χ*² = 5.04, *p* = 0.025). No statistically significant differences between the academic-stage groups were observed for daily water-consumption frequency (*χ*² = 0.003, *p* = 0.956) or the recommended walking-distance standard (*χ*² = 2.61, *p* = 0.106) (Table 1).

**Table 1:** Comparison of correct responses to knowledge questions by gender and academic stage.

Variable	Group	Correct *n* (%)	Incorrect *n* (%)	*χ*2/*p*-value
Daily Consumption Frequency	Female	66 (59.5)	45 (40.5)	2.37/0.124
	Male	67 (49.6)	68 (50.4)	
	Intern	31 (54.4)	26 (45.6)	0.003/0.956
	Pre-clinical	102 (54.0)	87 (46.0)	
Required Daily Intake	Female	9 (8.1)	102 (91.9)	2.64/0.105
	Male	20 (14.8)	115 (85.2)	
	Intern	15 (26.3)	42 (73.7)	15.06/ < 0.001
	Pre-clinical	14 (7.4)	175 (92.6)	
Walking Distance	Female	40 (36.0)	71 (64.0)	0.03/0.871
	Male	50 (37.0)	85 (63.0)	
	Intern	26 (45.6)	31 (54.4)	2.61/0.106
	Pre-clinical	64 (33.9)	125 (66.1)	
Water Conservation	Female	54 (48.6)	57 (51.4)	0.006/0.938
	Male	65 (48.1)	70 (51.9)	
	Intern	35 (61.4)	22 (38.6)	5.04/0.025
	Pre-clinical	84 (44.4)	105 (55.6)	

Water Consumption Behaviour Scale subdimension and total scores were compared between the two independent academic-stage groups.

Internship students had significantly higher scores than pre-clinical students in the Water Awareness (11.98 ± 2.10 vs. 9.71 ± 2.78; *p* < 0.001) and Personal and Social Responsibility (9.08 ± 1.97 vs. 6.77 ± 3.00; *p* < 0.001) subscales. In contrast, pre-clinical students scored significantly higher in the Water Consumption (13.79 ± 3.39 vs. 9.38 ± 2.19; *p* < 0.001), Water Pollution (11.92 ± 2.44 vs. 8.61 ± 2.18; *p* < 0.001), and Water Management (9.93 ± 2.89 vs. 8.66 ± 2.70; *p* = 0.004) subscales. Consequently, pre-clinical students also had significantly higher total Water Consumption Behaviour Scale scores than internship students (52.14 ± 10.43 vs. 47.73 ± 7.51; *p* = 0.001). Thus, the direction of the between-group association varied across subdimensions. This pattern represents differing cross-sectional associations across the measured subdimensions (Table 2).

**Table 2:** Comparison of water consumption behaviour scale scores by academic stage.

Sub-Dimensions	Intern (*n* = 57) (Mean ± SD)	Pre-clinical (*n* = 189) (Mean ± SD)	Mean Difference (Intern−Pre-clinical) (95% Confidence Interval)	Effect Size (Cohen’s d)	*p*-value
Water Awareness	11.98 ± 2.10	9.71 ± 2.78	2.27 (1.59 to 2.95)	0.86*	<0.001
Responsibility	9.08 ± 1.97	6.77 ± 3.00	2.32 (1.64 to 2.99)	0.83*	<0.001
Water Consumption	9.38 ± 2.19	13.79 ± 3.39	−4.41 (−5.16 to −3.65)	−1.39*	<0.001
Water Pollution	8.61 ± 2.18	11.92 ± 2.44	−3.31 (−3.99 to −2.64)	−1.39**	<0.001
Water Management	8.66 ± 2.70	9.93 ± 2.89	−1.27 (−2.10 to −0.44)	−0.44**	0.004
Total Score	47.73 ± 7.51	52.14 ± 10.43	−4.41 (−6.88 to −1.93)	−0.45*	0.001

Values are presented as mean ± SD. Mean differences are shown with 95% confidence intervals (95% CI), and effect sizes are reported as Cohen’s d. Homogeneity of variances was assessed using Levene’s test.

*Welch’s *t*-test was applied when equal variances were not assumed; otherwise, **Student’s independent-samples t-test was used.

Female students had higher Water Awareness and Water Pollution scores than male students (*p* < 0.01) and their total score was also higher (*p* = 0.004). No statistically significant gender differences were observed in the Water Consumption, Water Management, or Personal and Social Responsibility subdimensions (Table 3).

**Table 3:** Comparison of water consumption behaviour scale scores by gender.

Sub-Dimensions	Female (*n* = 111) (Mean ± SD)	Male (*n* = 135) (Mean ± SD)	Mean Difference (95% Confidence Interval)	Effect Size (Cohen’s d)	*p*-value
Water Awareness	10.82 ± 2.67	9.75 ± 2.82	1.07 (0.38 to 1.77)	0.39	0.003
Water Pollution	11.71 ± 2.58	10.70 ± 2.83	1.01 (0.33 to 1.69)	0.37	0.004
Water Consumption	13.25 ± 3.57	12.37 ± 3.70	0.87 (−0.04 to 1.79)	0.24	0.063
Water Management	9.94 ± 2.87	9.39 ± 2.90	0.55 (−0.18 to 1.28)	0.19	0.137
Responsibility	7.41 ± 3.12	7.22 ± 2.83	0.19 (−0.56 to 0.95)	0.06	0.614
Total Score	53.15 ± 9.70	49.45 ± 9.95	3.70 (1.22 to 6.18)	0.38	0.004

*Mean differences are reported with 95% confidence intervals (95% CI), and effect sizes are expressed as Cohen’s d. Homogeneity of variances was assessed using Levene’s test. Because equal variances were confirmed, Student’s independent-samples t-test was applied.

## Discussion

This study identified different patterns of water-related knowledge and scale scores in two independent academic-stage groups. Year 6 status was associated with higher correct-response rates for selected knowledge items, whereas the direction of the associations varied across the scale subdimensions.

Year 6 students had higher correct-response rates than Year 3 students for recommended daily water intake and water-conservation methods. Previous studies have also reported differences between students at different academic stages (4, 6). These between-group differences represent cross-sectional associations with academic stage. Because the study compared independent cohorts assessed at different times, the findings indicate cross-sectional associations between academic stage and the selected knowledge items. From a bioethical perspective, these cross-sectional differences are consistent with the view that water stewardship is an important component of distributive justice and planetary health (4, 9–11).

This interpretation is consistent with previous literature. Chase et al. emphasized that planetary health and sustainable healthcare remain insufficiently integrated into many medical curricula despite increasing recognition of their importance (6). Similarly, Barna et al. reported that scientific and clinical competencies receive greater curricular representation than environmental sustainability competencies in undergraduate medical education (4). In the present study, Year 6 students had higher correct-response rates for selected knowledge items, whereas Year 3 and Year 6 students had higher scores on different scale subdimensions. Given the analytical cross-sectional design, these findings represent associations between academic stage and the measured outcomes (4, 6, 12). Previous studies have reported higher sustainability awareness in educational settings where planetary health and environmental sustainability are explicitly represented in the curriculum. These reports highlight planetary health and environmental sustainability as relevant topics for curriculum development. However, the present study did not measure curricular exposure or evaluate curriculum effectiveness. Therefore, it cannot determine whether curricular experiences explain the observed differences between the two independent cohorts (13, 14).

From the perspective of distributive justice, access to clean water is recognized as a fundamental human right, as emphasized in the United Nations Sustainable Development Goals (9). In the present study, many students were unable to correctly identify WHO water service standards. Similar findings have been reported in studies demonstrating limited understanding of global environmental determinants of health among medical students (15). Taken together with previous literature, these findings may be interpreted as indicating that technical knowledge alone may not be sufficient to prepare future physicians to address global water-related health challenges. From the perspective of distributive justice, equitable access to safe water is a fundamental human right and an essential component of health equity (9, 16).

Although Year 6 students had a higher correct-response rate for the water-conservation item, 38.6% answered this item incorrectly. Previous studies have reported greater curricular representation of clinical and scientific competencies than of environmental sustainability (4, 6). In the present sample, the pattern of correct responses to the knowledge items was not uniform across the Water Consumption Behaviour Scale subdimensions. From a bioethical perspective, the Principle of Beneficence proposed by Beauchamp and Childress emphasizes that physicians should promote well-being while avoiding harm (17). Within the framework of planetary health, this responsibility has been interpreted as extending beyond the immediate needs of individual patients to include the protection of environmental resources essential for the health of present and future generations (10). Accordingly, beneficence may be considered within a broader ecological framework that includes the protection of water resources essential to human and ecosystem health (10, 17, 18). Water ethics links water use and management with human dignity, equity, solidarity, the common good, responsible stewardship, public health, and obligations to future generations (18). Previous literature has highlighted the importance of planetary health and environmental sustainability in medical education (4, 6, 13, 14, 19). However, the present findings should be interpreted as cross-sectional associations between independent academic-stage groups.

The groups showed different patterns across the subscale and total scores. Internship students had significantly higher Water Awareness and Responsibility scores, whereas pre-clinical students had significantly higher Water Consumption, Water Pollution, Water Management, and total scale scores. This pattern indicates that the differences between the groups were not consistent across all dimensions of the Water Consumption Behaviour Scale and should therefore be interpreted separately for each subdimension as well as for the overall score. The mean total scale score was 52.14 among pre-clinical students and 47.73 among internship students (*p* = 0.001). These findings represent cross-sectional associations between academic stage and the measured outcomes. Longitudinal studies are needed to clarify the temporal relationship between academic stage and water-related outcomes (20).

The scale’s five subdimensions—water awareness, responsibility, consumption, pollution, and management—reflect distinct aspects of water-related knowledge, attitudes, and behaviours relevant to preventive medicine and environmental bioethics. As discussed above, the two groups showed different patterns across the scale subdimensions; therefore, the findings should not be interpreted as indicating a uniform difference in environmental awareness. From the perspective of Hans Jonas's Principle of Responsibility, physicians have an ethical obligation to protect the environmental conditions that sustain human life, including the responsible stewardship of water resources for future generations (21). Because the study compared independent cohorts assessed in different calendar years, the observed differences represent cross-sectional associations between academic stage and the measured subdimensions (12). Virtue Ethics also provides a normative framework for considering environmental responsibility alongside clinical competence (22).

No statistically significant gender differences were observed in correct responses to the water-consumption knowledge questions in the overall sample. Female students had higher Water Awareness, Water Pollution, and total scale scores than male students, whereas Water Consumption, Water Management, and Personal and Social Responsibility scores did not differ significantly by gender. Thus, the associations with gender were not consistent across the measured outcomes. Because the study did not assess factors that might explain these associations, their underlying reasons cannot be determined. From the perspective of Virtue Ethics, medical practice emphasizes the cultivation of professional character and practical wisdom in addition to technical proficiency (22). Within this theoretical framework, environmental responsibility may be considered alongside clinical competence as a relevant dimension of medical professionalism (22). Longitudinal studies following the same students over time are needed to determine whether the observed associations persist and to identify factors associated with these patterns. As global environmental challenges continue to evolve, previous literature emphasizes the importance of planetary health and environmental responsibility within contemporary medical education (11). The observed knowledge and attitude patterns identify water awareness, responsibility, consumption, pollution, and management as relevant topics for undergraduate medical curricula.

## Limitations

This study has several limitations. First, the analytical cross-sectional design does not permit causal inference or assessment of individual changes over time. Second, the study was conducted at a single medical school, which may limit the generalizability of the findings. Third, participants were recruited using a convenience sampling approach based on voluntary participation, introducing the possibility of selection bias. Fourth, the findings relied on self-reported responses, which may be subject to recall and social desirability bias. Finally, because the two independent cohorts were assessed in different calendar years, temporal and cohort-related factors cannot be entirely excluded when interpreting the observed differences. Future multicenter longitudinal studies are needed to confirm these findings.

## Conclusion

Academic stage was associated with distinct water-related knowledge and attitude patterns. Year 6 students had higher correct-response rates for recommended daily water intake and water-conservation methods and higher Water Awareness and Personal and Social Responsibility scores. Year 3 students had higher Water Consumption, Water Pollution, Water Management, and total scale scores. Because the study compared two independent cohorts assessed at different calendar times, the findings represent cross-sectional associations between academic stage and the measured outcomes. Longitudinal and multicentre studies are needed to examine temporal patterns and possible explanatory factors.

## Data Availability

The original contributions presented in the study are included in the article/supplementary material, further inquiries can be directed to the corresponding author.
